# P02.61. Does yogic breathing (pranayama) improve cardiovascular control?

**DOI:** 10.1186/1472-6882-12-S1-P117

**Published:** 2012-06-12

**Authors:** S Bertisch, C Tan, A Ahn, J Taylor

**Affiliations:** 1 Beth Israel Deaconess Medical Center, Boston, USA; 2 Spaulding Rehabilitation Hospital, Harvard Medical School, Boston, USA; 3 Massachusetts General Hospital, Harvard Medical School, Boston, USA

## Purpose

One proposed mechanism by which meditative practices improve cardiovascular health is via shifting sympathovagal balance. Yet few studies have examined the effects of these practices upon autonomic cardiovascular control. In this context, we hypothesized that yogic breathing differentially impacts heart period (RR-interval) and systolic blood pressure fluctuations compared with paced breathing at 15 breaths/minute.

## Methods

We enrolled five healthy advanced yoga practitioners from diverse traditions. We continuously measured heart rate, beat-by-beat blood pressure, respiratory rate, inspiratory volume, and end-tidal CO_2_ during paced breathing (10 minutes) and yogic breathing (20 minutes). For yogic breathing, practitioners performed a deep breathing pranayama of their choice. We performed standard time and frequency domain analyses.

## Results

In this group of practitioners, yogic breathing was characterized by a mean respiratory rate of 4.32 ± 1.87 breaths/min, mean minute ventilation of 6.09 ± 4.17 L/min, and mean end-tidal CO_2_ of 39.98 ± 7.07 mmHg compared with a mean respiratory rate of 15.0 ± .60 breaths/min, minute ventilation of 8.66 ± 2.25 L/min, and mean end-tidal CO_2_ of 32.68 ± 5.22 mmHg during paced breathing. The strength of the relationship (coherence) between respiration and heart period and respiration and systolic blood pressure did not differ between breathing patterns (p=0.74 and p=0.14). However, we found increased gain between respiration and heart period fluctuations (400.00 ± 154.19 msec/L vs 36.65 ± 10.32 mmHg/L, p=0.02) and between respiration and systolic blood pressure fluctuations (15.97 ± 9.32 msec/L vs 1.68 ± 0.68 mmHg/L, p=0.02) during yogic breathing compared with paced breathing.

## Conclusion

Yogic breathing was associated with increased gain compared with paced breathing. Whether the larger gain relation between respiration and cardiovascular fluctuations during yogic breathing represents baroreflex modulation or is due to enhanced entrainment through repetitive practice remains to be established. However, these preliminary findings may explain some of yoga’s reported cardiovascular benefits.

